# Multifractal Downscaling of Rainfall Using Normalized Difference Vegetation Index (NDVI) in the Andes Plateau

**DOI:** 10.1371/journal.pone.0168982

**Published:** 2017-01-26

**Authors:** L. A. Duffaut Espinosa, A. N. Posadas, M. Carbajal, R. Quiroz

**Affiliations:** 1 Electrical and Biomedical Engineering Department, University of Vermont, Burlington, Vermont, United States of America; 2 Production Systems and the Environment Division, International Potato Center (CIP), Lima, Perú; 3 World Agroforestry Centre (ICRAF), Nairobi, Kenya; CNRS, FRANCE

## Abstract

In this paper, a multifractal downscaling technique is applied to adequately transformed and lag corrected normalized difference vegetation index (NDVI) in order to obtain daily estimates of rainfall in an area of the Peruvian Andean high plateau. This downscaling procedure is temporal in nature since the original NDVI information is provided at an irregular temporal sampling period between 8 and 11 days, and the desired final scale is 1 day. The spatial resolution of approximately 1 km remains the same throughout the downscaling process. The results were validated against on-site measurements of meteorological stations distributed in the area under study.

## Introduction

Rainfall information is one of the most important inputs to agricultural models in areas of difficult accessibility such as the Andean high plateau. To obtain information at high spatial resolution of phenomena such as rainfall and temperature variation, researchers employ indirect forms of rainfall information. It is usually the case that several meteorological stations are spread out to cover the study area, but only the data from meteorological stations is not enough to amount for the spatial variability of rainfall, and keeping the stations running is often expensive and unfeasible in certain regions, for example, of the Andes. One alternate source of spatial variability is the so-called *normalized difference vegetation index* (NDVI). In the range from 200 to 1200 mm per year, NDVI has been reported to show a linear relationship with respect to rainfall [1, 2]. The limit for such linearity corresponds to areas with low annual precipitations [3]. Above 1200 mm per year, NDVI appears to *saturate*. Therefore, the index increases slowly when rainfall increases or reaches a constant plateau. However, NDVI correspondence to rainfall only makes sense at a temporal resolution in the order of 8 to 10 days periods due to its intrinsic smoothness [4]. Therefore, for agriculture applications NDVI requires to be downscaled in time, so that intermittency (generation of zero rainfall values) is added and a useful temporal resolution is achieved, say daily.

To process NDVI information as an indirect measure of rainfall, it is necessary to overcome several challenges. For example, NDVI does not provides clear information about rainfall intermittency, its response is cumulative (amounting for its smoothness), its response is almost always delayed many days after rainfall has fallen in the region, and there is a need for auxiliary information about the long term statistics of rainfall in the regions where there are no meteorological stations so that a proper transformation from NDVI measure to rainfall measurements is performed.

The high variability of rainfall suggests statistical downscaling as an appropriate technique for temporal downscaling of NDVI. In particular, it is desired to exploit the scaling behavior of the phenomena, thus a multifractal technique is chosen for the task. Among multifractal techniques, there are canonical and microcanonical procedures [5]. The cumulative nature of NDVI suggests the use of a microcanonical approach since roughly speaking the dissagregation process preserves exactly the rainfall amount in every step of the dissagregation process (mass/energy conservation). That is, the sum of weights of the random generator of the statistical microcanonical temporal downscaling procedure is exactly 1.

The goal of this manuscript is threefold. First, obtain rainfall from spatio-temporal NDVI information with an 8 day period temporal resolution (because of the dyadic nature of the cascading procedure). This requires using auxiliary information coming from some meteorological stations in order to estimate the intermittency and scale factor for the NDVI transformation. Then the time between rainfall and the NDVI response is computed and used to translate the the NDVI time series. Finally, the multifractal temporal downscaling procedure is applied to the transformed NDVI information in order to obtain an estimation of a 1 day period rainfall information.

## NDVI Data

**Fig 1:**
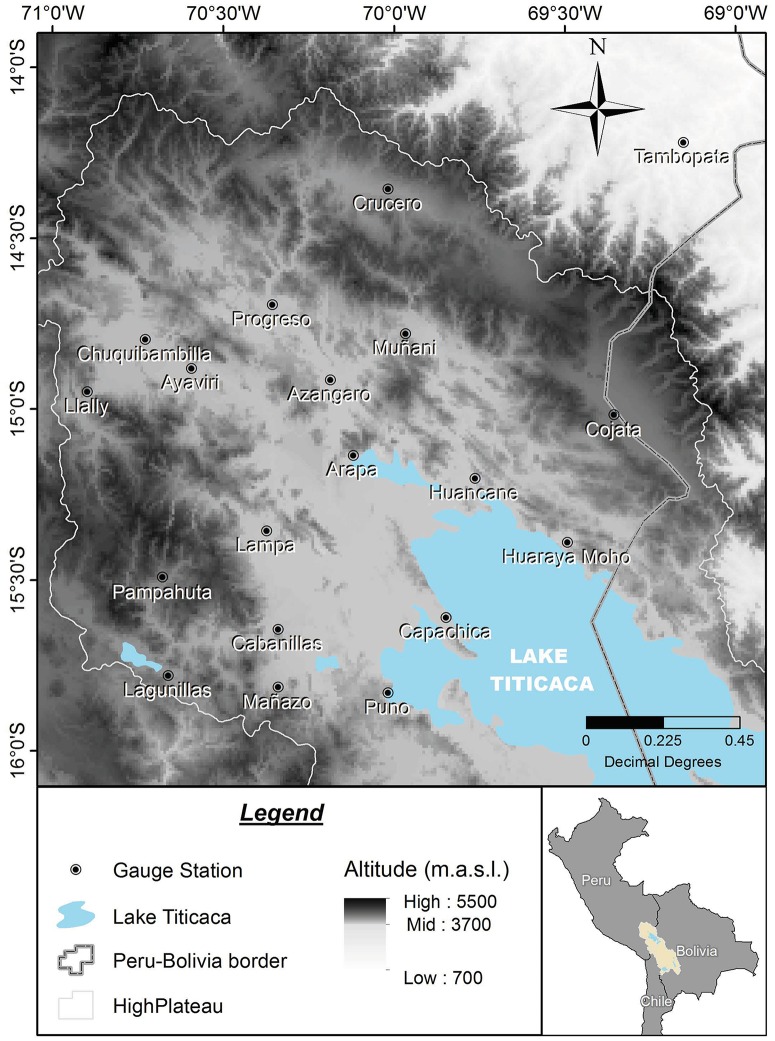
Study region.

The NDVI dataset (in S1 File) consists of 288 (dekad) composite images(225 × 225 pixels)with an approximate resolution of 1km corresponding to the area shown in Fig 1. This NDVI is derived from the vegetation instruments SPOT-4 and SPOT-5 over the 46 time period starting in January 1999 and ending in December 2006. The period from 47 January 2007 to December 2007 is also considered in this work for correction 48 purposes [2]. The spectral and spatial resolution of the vegetation instruments is the 49 same. The spectral band 0:61-0:68 mm corresponding red and the band 0:78-0:89 mm 50 corresponding to near-infrared (NIR) were used to compute the NDVI index by employing the standard formula

$$NDVI=\frac{NIR-RED}{NIR+RED}.$$ (1)

The final product has a spatial resolution of ∼ 1 km. The above formula for the NDVI index restricts the values to be in the interval [−1, 1]. In addition, the NDVI index is geometrically and radiometrically corrected producing the *S*10 NDVI product [6]. The 288 days (dekad) data set were defined according to the civil calendar. Every month was divided into 3 pieces: from the 1st day to the 10th; from the 11th to the 20th; and from the 21st to the end of each month. Each month therefore produces 3 NDVI data points per month. This NDVI information, however, is not appropriate for downscaling purposes since the sampling period of the time series is not uniform. Using the smoothness assumption on NDVI data [4], a simple spline interpolation was applied to NDVI in order to standardize the time scale. In this manner, NDVI becomes an evenly spaced (in time) signal. For the sake of simplicity, the time scale of 8 days was chosen so that a dyadic cascade procedure can be performed in subsequent sections.

NDVI information is such that its value at time *t* can be considered as the cumulated effect of an “input process” in the time interval [*t*−*T*/2, *t* + *T*/2], where *T* is the size of the time interval. In our case, the input process is rainfall. Therefore, its disaggregation preserves the total NDVI measure value. That is,

$$\begin{array}{c} NDVI\left(\left[t-T/2,t+T/2\right]\right)=NDVI\left(\left[t-T/2,t_{1}\right]\right)+NDVI\left(\left[t_{1},t+T/2\right]\right) \end{array}$$

for *t*−*T*/2 < *t*_1_ < *t* + *T*/2. Since in our application *T* is fixed, then *NDVI*([*t*−*T*/2, *t* + *T*/2]) is denoted as *NDVI*(*t*). In addition, NDVI responds after rainfall has fallen [6]. This lag between the NDVI signal and rainfall measurements can be better understood when considering that one of the primordial uses of NDVI is estimating the biomass index in a region [7]. Thus, the lag in the NDVI signal is the latency time that takes between rainfall reaching the ground and the time changes in the biomass index are registered in the red and infrared frequencies, which then are used to compute Eq (1). In addition, the rainfall information requires some knowledge of the probability of zero rain, namely, the rainfall intermittency. Thus, the rainfall measurements obtained from NDVI, say *NDVI*_*rain*_, values can be modeled as

$$\begin{array}{c} NDVI_{rain}\left(t\right)=F\left(NDVI\left(t\right),lag,v\right), \end{array}$$ (2)

where *F* is a function of the shift in time *lag* and the parameter *v* associated to the rainfall intermittency [2]. We will see later that the function *F* amounts to a vertical traslation of NDVI as well as a linear resizing coming from auxiliary information.

NDVI has high spatial resolution (∼1 km) when compared to the usual satellite data (∼30 km or more). Therefore, this fact makes NDVI a great source of spatial information if converted to rainfall measurements. However, NDVI has a smooth response, and therefore it dresses the intermittency of rainfall in time. It is thus that NDVI only makes sense as a source of rainfall information at coarse temporal resolution, for example 8 or 10 day period temporal resolution. Information about the intermittency probability is needed for downscaling NDVI to a daily resolution, so an auxiliary source of information is needed for such task. Once this intermittency probability is known, the downscaling process inherently generates zero value measurements through a multiplicative cascade procedure as described in the next section. Unfortunately, information at high resolutions is scarce or nonexistent in the Andean high plateau, however on-site meteorological stations measurements (locations of such stations are shown in Fig 1 and Table 1) can be used to generate an approximate rainfall field by employing the relationship between rainfall and elevation [8]. The stations measurements are provided in S1 Table and the corresponding locations in the 225 × 225 grid are given in S2 Table. For this purpose, the thin-plate smoothing spline algorithm implemented in the ANUSPLIN 4.36 package [9, 10] is used to generate such rainfall fields, which consider in addition to measured rainfall the latitude, longitude, and elevation of the area [11]. The method was chosen due to its higher accuracy compared to other methods in areas similar to the Andes high plateau [8, 12–14]. Also, several climate products such as WorldClim ([8], http://www.worldclim.org) and IWMI Climate Atlas/CRU gridded data ([15], http://www.iwmi.org, http://www.cru.uea.ac.uk) have successfully applied the methodology that the ANUSPLIN package provides.

**Table 1:** Weather station locations and altitudes.

Weather station	Longitude (degrees)	Latitude (degrees)	Altitude (m.a.s.l.)
Arapa	-70.12	-15.14	3920
Ayaviri	70.59	-14.88	3920
Azángaro	-70.19	-14.91	3863
Cabanillas	-70.35	-15.64	3890
Capachica	-69.84	-15.62	3819
Chuquibambilla	-70.73	-14.80	3910
Cojata	-69.36	-15.02	4344
Crucero Alto	-70.02	-14.36	4130
Huancané	-69.76	-15.20	3860
Huaraya Moho	-69.49	-15.39	3890
Lagunillas	-70.66	-15.77	4250
Lampa	-70.37	-15.36	3900
Llally	-70.90	-14.95	4111
Mañazo	-70.34	-15.81	3942
Muñani	-69.97	-14.78	4119
Pampahuta	-70.68	-15.49	4320
Progreso	-70.36	-14.69	3965
Puno	-70.02	-15.82	3840
Tambopata	-69.15	-14.22	1340

## Downscaling Method

A multiplicative random cascade divides a seed rainfall measurement and probabilistically assign to a subdivision of the seed area new rainfall measurements. the subdivisions are characterized by the *branching number* *b*, which in the temporal case (one dimensional) is *b* = 2. After *n* subdivision, one denotes the *i*-th interval (out of *i* = 1, …, *b*^*n*^ intervals at level *n*) by $\Delta_{n}^{i}$ and define *λ*_*n*_ = *b*^−*n*^ as the dimensionless spatial scale. It then follows that the mass in subdivision $\Delta_{n}^{i}$ is:

$$\begin{array}{c} \rho_{n}\left(\Delta_{n}^{i}\right)=\rho_{0}\lambda_{n}\prod_{j=1}^{n}W_{j}\left(i\right) \end{array}$$ (3)

for *i* = 1, 2, …, *b*^*n*^, *n* > 0, *ρ*_0_ is the initial rainfall measurement at *n* = 0 and *W*_*j*_ is the *cascade generator* at level *j*.

### Multifractal Cascade Model

The cascade is called *microcanonical* in that mass is preserved exactly at every level of the multiplicative cascade (in contrast the *canonical model* preserves means; see [5]). In this case, the random variable *W*_*n*_ (the cascade generator at level *n*) is constrained to preserve exactly the measurement in the previous cascade level. That is, every new level consisting on subdividing every measurement into *b* new measurements satisfies

$$\begin{array}{c} \sum_{k=1}^{b}W_{n}\left(b\left(i-1\right)+k\right)=1\;\text{for}\;i=1,2,\ldots ,b^{n-1} \end{array}$$ (4)

As in the *β*-lognormal model introduced in [16], intermittency (or zero values generation) in the microcanonical model is introduced by allowing *W*_*n*_(*i*) = 0 in a multiplicative manner. If the random generator is symmetric then the microcanonical model disaggregates every nonzero rainfall amount in the interval *i* at scale *n*−1 into *b* = 2 intervals at scale *n*. However, two situations may occur: One is that intermittency can emerges in one interval only at the scale *n* with probability *p*_0, *w*_, i.e.,

$$\begin{array}{c} P\left(W_{n}\left(j\right)=0\vee W_{n}\left(j+1\right)=0\right)=p_{0,w}. \end{array}$$

The other situation is that zero measurements do not occur, which imply that the new intervals *j* and *j* + 1 have the value of the random generator in the open interval (0, 1). The reader is referred to [17, 18] for continuous models recently proposed to address intermittency. In particular, one can characterize the random generator *W*_*n*_ by associating its probability distribution to that of the so-called *breakdown coefficients* [19–21].

### Breakdown Coefficients

Let *R* be a *D*-dimensional random field. For a *D*-dimensional box Δ_*T*_ of size *T*^*D*^, the breakdown coefficient of a box Δ_*τ*_ of size *τ*^*D*^ inside Δ_*T*_ is the ratio between the aggregated measure *R*_*τ*_ over Δ_*τ*_ and the total rainfall *R*_*T*_ over Δ_*T*_. That is,

$$\begin{array}{c} W\left(\tau ,T\right)=\frac{R_{\tau }}{R_{T}}\;\text{for}\;\tau <T. \end{array}$$ (5)

**Fig 2:**
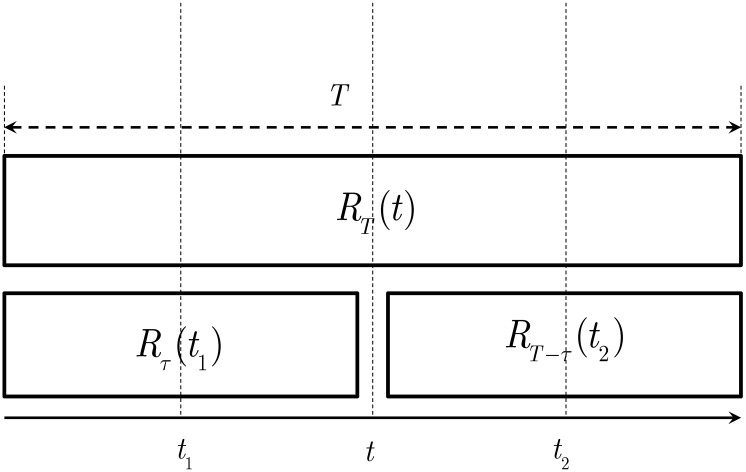
Breakdown coefficient.

Since in this paper *D* = 1 and the branching parameter in Eq (3) is *b* = 2, the interval Δ_*T*_ centered at *t* is breakdown into two pieces with lengths *τ* and *T*−*τ* and respectively centered at *t*_1_ and *t*_2_ as shown in Fig 2. Therefore, for every interval of length *T*, one has the breakdown parameters $W\left(\tau ,T\right)=\frac{R_{\tau }\left(t_{1}\right)}{R_{T}\left(t\right)}$ and $W\left(T-\tau ,T\right)=\frac{R_{\tau }\left(t_{1}\right)}{R_{T}\left(t\right)}$.

Note that 0 ≤ *W*(*τ*, *T*) ≤ 1 and that it only depends on the branching parameter *b*. Picking *τ* = *T*/2, the breakdown parameters are computed at different scales by including the aggregation of the random field at a scale *λ*_*n*_ = *b*^−*n*^. Hereafter, the ℓ-th breakdown coefficient at scale *λ*_*n*_ is denoted as *W*_*n*_(*ℓ*) for *ℓ* ∈ {1, …, *b*^*n*^}. Therefore, at scale *λ*_*n*_ and using the indexation in Eq (4), the *ℓ* = *b*(*i*−1) + *k* breakdown coefficient is

$$\begin{array}{c} W_{n}\left(b\left(i-1\right)+k\right)=\frac{R_{T/2,\lambda_{n}}}{R_{T,\lambda_{n}}}=\frac{\rho_{n}\left(\Delta_{n}^{b(i-1)+k}\right)}{\rho_{n}\left(\Delta_{n}^{b(i-1)+1}\right)+\rho_{n}\left(\Delta_{n}^{b(i-1)+2}\right)}, \end{array}$$

where *i* ∈ {1, …, 2^*n*−1^}, *k* ∈ {1, 2}, and the intervals $\Delta_{n}^{b(i-1)+1}$ and $\Delta_{n}^{b(i-1)+2}$ are subdivisions of an interval $\Delta_{n-1}^{i^{\prime }}$ for *i*^′^ ∈ {1, …, 2^*n*−1^}.

From the theory of self-similar fields in [20], one has that Eq (5) satisfies

$$\begin{array}{c} E\left[W{\left(\tau ,T\right)}^{q}\right]\propto {\left(T/\tau \right)}^{K(q)} \end{array}$$

where *K*(0) = *K*(1) = 0 and *K*(*q*) is the *moment scaling exponent* function in [22, 23]. Note also that Eq (5) have the property that

$$\begin{array}{c} E\left[W{\left(\tau ,T\right)}^{q}\right]=E\left[W{\left(\tau ,\nu \right)}^{q}\right]E\left[W_{n}{\left(\nu ,T\right)}^{q}\right] \end{array}$$

with *τ* < *ν* < *T*. Taking the logarithm of the breakdown coefficients *x*(*τ*, *T*) ≔ log*W*(*τ*, *T*), it then follow for *τ* < *τ*_1_ < … < *τ*_*s*_ < *T*} that

$$\begin{array}{c} x\left(\tau ,T\right)=x\left(\tau ,\tau_{1}\right)+x\left(\tau_{1},\tau_{2}\right)+\cdots +x\left(\tau_{s},T\right). \end{array}$$

If the terms in the right-hand-side of the above equation are i.i.d random variables whose distribution depends only on (*τ*/*T*)^1/*s*^, then the p.d.f of the random variables *p*(*x*, (*τ*/*T*)^1/*s*^) is the probability distribution of the logarithm of the breakdown parameters. The p.d.f. *p*(*x*, *τ*/*T*) is the *s*-fold convolution of *p*(*x*, (*τ*/*T*)^1/*s*^), which characterizes the scaling behavior of the field in terms of the parameters of *p*(*x*, *τ*/*T*) [19]. Furthermore, following [20 Section 2], the moment generating function $\psi \left(q,\tau /T\right)=L\left\{p\left(x,\tau /T\right)\right\}={\left(\tau /T\right)}^{-\chi (q)}$, where L{·} denotes the Laplace transform in the variable *q*, is used to obtain the relation

$$\begin{array}{c} K(q)=qD+\chi (q). \end{array}$$

Thus, the breakdown coefficients are a tool that can be employed to study the moment scaling function that characterizes a random field since the breakdown coefficients density function does not vary through scale. This case is called *self-similar*. Nevertheless, it was observed in [19] that the probability density of the breakdown coefficients of rainfall have the same shape but their variance increases with the resolution. This is characteristic of self-affine random fields. For these fields, the computation of the breakdown coefficients is exactly the same, but the probability density function parameter follow a power law with respect to the scale. For instance, if *a* is such a parameter, then

$$a=a(0){\left(\frac{\tau }{T}\right)}^{-H},$$ (6)

where *a*(0) and *H* are computed empirically from the breakdown coefficients histograms. This case is thus called *self-affine* [19, 21].

### The Beta Distributed Random Generator

Classically, rainfall time series have been fitted to Gamma distributions [24]. While any infinitely divisible probability distribution can be used in the theory described in the previous sections (e.g., any *α*-stable distribution [23]), the generation of random numbers satisfying Eq (4) is not straightforward. However, a pragmatic choice for a probability distribution of *W*_*n*_ is the Beta distribution. This is due to its nice analytic properties. The Beta distribution is given by

$$\begin{array}{c} p\left(r\right)=\frac{1}{B(a,c)}r^{a-1}{\left(1-r\right)}^{c-1}, \end{array}$$ (7)

where $B\left(a,c\right)=\int_{0}^{1}x^{a-1}{\left(1-x\right)}^{c-1}\;dx$ is the well-known *Beta function*. For the random generator *W*_*n*_ probability distribution *p*_*W*_*n*__ is given by the symmetric Beta distribution. That is, *p*_*W*_*n*__ is Eq (7) when *a* = *c*. Note that if *a* = 1, then it conveys a uniform distribution; on the other hand if *a* > 1, then the distribution is located around *E*(*W*) and is bell shaped. Also, increasing or decreasing the only parameter *a* has an effect on the width of the distribution, which is desired in the characterization of the breakdown coefficients distributions with respect to the scale. Moreover, from the properties of the Beta function, the mean and variance of a Beta distribution with parameters *a* and *c* are

$$\begin{array}{c} E\left(W\right)=\frac{a}{a+c}\;\text{and}\;Var\left(W\right)=\frac{ac}{\left(a+c+1\right){\left(a+c\right)}^{2}}, \end{array}$$

respectively. If *a* = *c*, then

$$\begin{array}{c} E\left(W\right)=\frac{1}{2}\;\text{and}\;Var\left(W\right)=\frac{1}{4(2a+1)}. \end{array}$$

Thus,

$$\begin{array}{c} a=\frac{1}{8Var(W)}-0.5, \end{array}$$

which can be used directly to compute the generator *W*_*n*_ distribution parameter *a* straight from the variance of the breakdown coefficient histograms. In this paper, a multifractal downscaling technique is applied to adequately transformed and lag corrected normalized difference vegetation index (NDVI) in order to obtain daily estimates of rainfall in an area of the Peruvian Andean high plateau. This downscaling procedure is temporal in nature since the original NDVI information is provided at an irregular temporal sampling period between 8 and 11 days, and the desired final scale is 1 day. The spatial resolution of approximately 1 km remains the same throughout the downscaling process. The results were validated against on-site measurements of meteorological stations distributed in the area under study.

When the branching number is *b* = 2, the two values *w*_1_ and *w*_2_, generated by *W*_*n*_, must satisfy Eq (4) and have to be distributed according to Eq (7). Computing constrained random numbers is the main difficulty in the microcanonical downscaling formalism. Fortunately, as pointed out in [5, 21], the generation of the generation of Beta distributed random numbers satisfying Eq (4) is done by computing two Gamma distributed random numbers *x*_1_ and *x*_2_, having the same parameter *a* as the objective symmetric Beta distribution, so that *w*_1_ = *x*_1_/(*x*_1_ + *x*_2_) and *w*_2_ = *x*_2_/(*x*_1_ + *x*_2_) are Beta distributed random numbers satisfying the required conditions.

## Application to Data in the Andes

**Fig 3:**
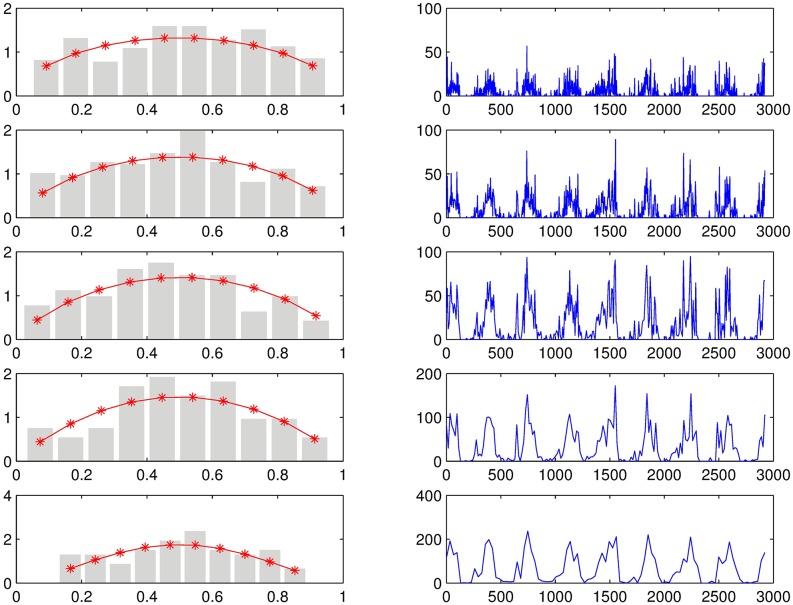
Histograms and their corresponding aggregation of the breakdown coefficients at different temporal resolutions (2, 4, 8, 16 and 32 days).

The microcanonical downscaling technique is first illustrated by applying the procedure to an 8 day period rainfall (aggregated) at Chuquibambilla Station (see Fig 1 and Table 1 for the exact location). That is, the rainfall time series at the station is aggregated from 1 day to 8 day period. In Fig 3, one can observe the aggregated series in the right and the corresponding histogram of breakdown coefficients on the left. Note how the variance reduces when the temporal resolution reduces (the day period augments and the distribution parameter increases).

**Fig 4:**
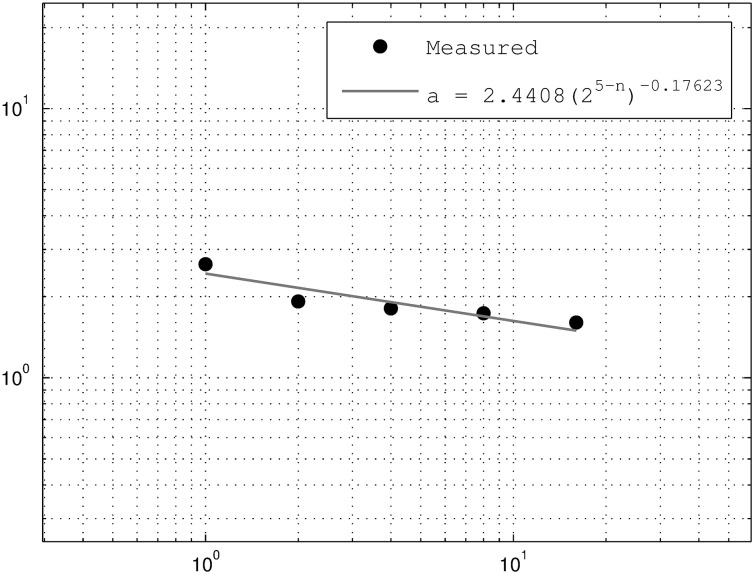
Beta p.d.f. parameter estimation from original time series.

**Fig 5:**
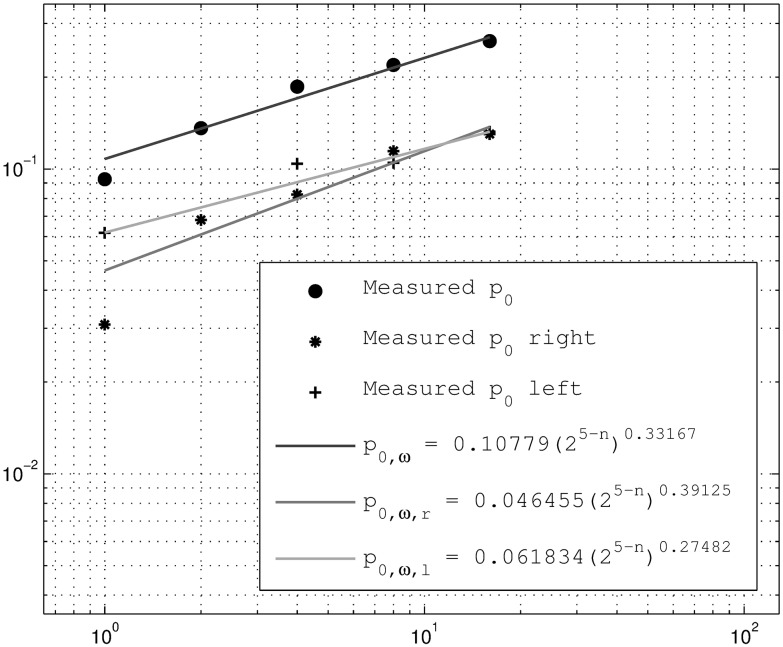
Intermittency parameter estimation from original time series (includes left and right interval intermittency probabilities).

**Fig 6:**
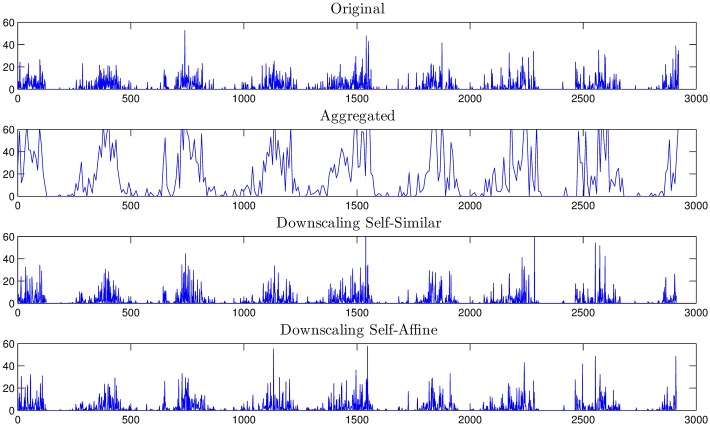
Downscaling comparison of the original rainfall series, the 3 level aggregated time series, and the downscaled rainfall for the self-similar and self-affine cases.

The parameter *a* is estimated directly from the variance of the weight distributions in the above histograms of the breakdown coefficients; see Fig 4. Similarly, the intermittency parameter *p*_0, *ω*_ is computed from the original time series by counting the zero weights on the breakdown coefficients; see Fig 5. Here it is observed that the zero probability of the left intervals are close to the one for the right intervals as expected. The downscaling of the aggregated time series (3 levels) for both downscaling cases, the self-similar and self-affine, can now be performed. The result of the downscaling procedure can be seen in Fig 6.

**Fig 7:**
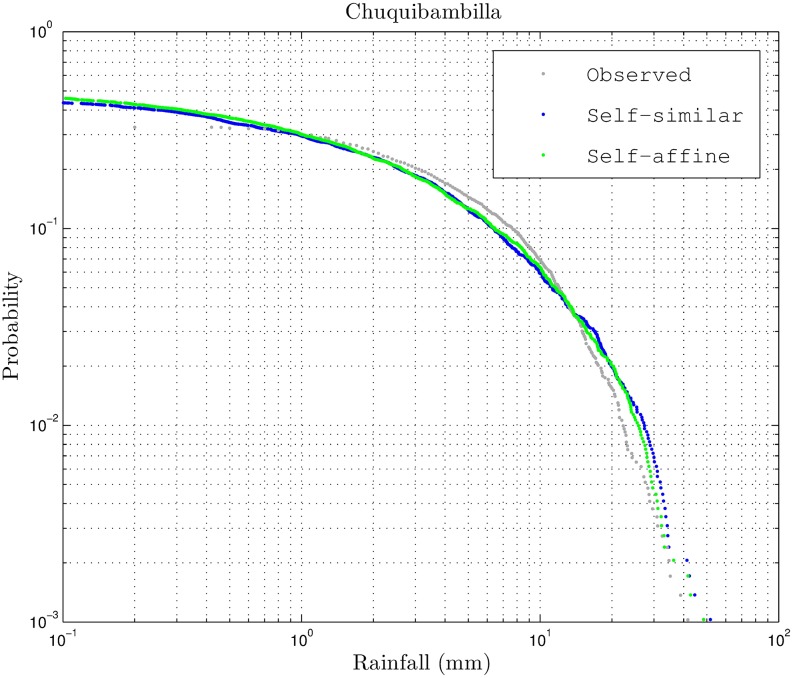
Exceedance probability of Chuquibambilla rainfall time series.

A comparison between exceedance probability plots of the observed rainfall and the downscaled rainfall is given in Fig 7. It means that the underlying probability behavior of both time series, observed and generated, are similar to each other as expected. In this case, the breakdown coefficients were computed directly from the observed rainfall, which also allowed obtaining the rainfall intermittency. This is not the case for locations in the Andes high plateau where there are no meteorological stations. In this manuscript, the same procedure will be applied to locations having only NDVI information. The auxiliary information will be obtained from a mesh of meteorological stations in the area under study. It is important to remark that the multifractality in the method lies in the characterization of the Beta distribution parameter *a*. In this case, a self-similar case will have a constant *a* for all scales (*H* = 0 in Eq (6)), and a self-affine case will have a non zero exponent *H* in the power law Eq (6). As shown by the breakdown coefficients histograms, the distribution appears to be self-affine from Fig 4. However, both self-similar and self-affine cases are applied in this manuscript.

### Correspondence Between NDVI and Rainfall Values

As mentioned in the previous section, the only information available in the area under study is NDVI. To be able to use NDVI as a measure of rainfall, one need to apply a suitable transformation. This transformation consists of two steps

1. Lag correction,
2. NDVI scale factor.

Both procedures require auxiliary information since NDVI needs a reference signal, in order to compute its lag with respect to rainfall, and to obtain an estimate of the intermittency and some simple statistics of rainfall at any location of the area under study. Here is where the ANUSPLINE estimates play a crucial role. That is, the ANUSPLINE time series will serve as the reference signal for the lag computation, and its mean and standard deviation at each point of the grid will be employed for the scaling of NDVI.

#### Lag Correction

Lag correction consists on calculating the lag time between the moment rainfall occurs and the time in which NDVI responds to it. For this one relies on harmonic analysis using a Fourier series decomposition of NDVI and the auxiliary ANUSPLINE information at a fixed point.

Fourier or harmonic analysis is a technique that decomposes a complex static signal into a summation of sines and cosines, where each wave is characterized by its corresponding amplitude and phase angle. Fourier analysis has been used successfully in the analysis of NDVI time series [6]. Assuming that the NDVI time series is described by a function *S*, then since the process is evidently periodic it can be described by the so-called *Fourier series*. That is, it can be described bu the following series

$$\begin{array}{c} S\left(t\right)=\frac{A_{0}}{2}+\sum_{n=1}^{\infty }A_{n}\cos\left(n\omega t\right)+\sum_{n=1}^{\infty }B_{n}\sin\left(n\omega t\right), \end{array}$$

where *A*_*n*_ and *B*_*n*_ are the usual Fourier coefficients.

The term $\frac{A_{0}}{2}$ is always equal to the mean value of *S*(*t*), and *ω* = 2*πf*_0_, where *f*_0_ is the characterizing frequency for all the waves in the decomposition. Since every cosine can be written as a phased sine the Fourier series, then a little algebra allows to write *S*(*t*) as

$$\begin{array}{c} S\left(t\right)=\frac{A_{0}}{2}\sum_{n=1}^{\infty }C_{n}\sin\left(n\omega t+\theta_{n}\right). \end{array}$$ (8)

For a discrete time signal as the one obtained from the NDVI index, the coefficients *C*_*n*_ can be obtained by using the Fast Fourier Transform (FFT). In our case, the FFT is used estimate the *C*_*n*_ coefficients of a signal comprised of 366 discrete NDVI data points corresponding to a temporal resolution of 8 days, which was obtained after resampling the original 288 NDVI data points. The FFT provides a complex vector having *A*_*n*_ coefficients in its real part and *B*_*n*_ coefficients in its imaginary part. Thus, the coefficients *C*_*n*_ of Eq (8) are derived from *A*_*n*_ and *B*_*n*_ by calculating the length of the vectors. The two main assumptions in order to use the FFT are: the signal must be sampled with a frequency of at least twice its bandwidth (Nyquist frequency), and both amplitude and phase of the signal should not vary significantly over time. Both requirements are satisfied by the resampled NDVI time series.

**Fig 8:**
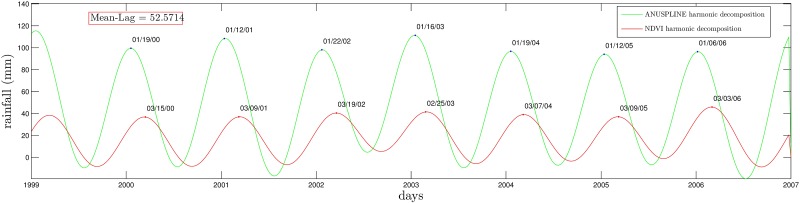
Harmonic comparison of NDVI and ANUSPLINE time series at Munani station.

Once NDVI and ANUSPLINE time series are represented by their Fourier series (truncated to 10 harmonics in this paper). The lag is simply computed taking the average of the day difference between peaks of NDVI and ANUSPLINE; see Fig 8.

**Fig 9:**
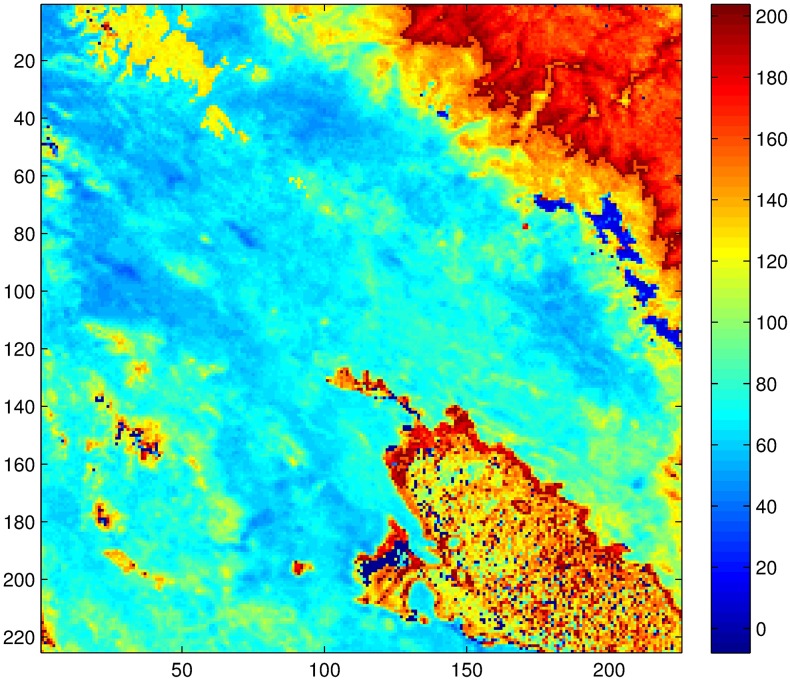
Mapping of lags for the region under study. The colorbar indicates lag in day units.

The procedure is repeated at all points in the study region. That is, the lag is computed for 225 × 225 locations. This gives the map of lags in Fig 9. The reader should notice that there are some areas in the region in which either there is a water body or rainforest in which the threshold for the linearity between NDVI and rainfall is exceeded (recall that such threshold is about 1200 mm in a year), and the computed lag is unreliable or simply makes no sense at all on those locations due to saturation.

#### NDVI scale factor

**Fig 10:**
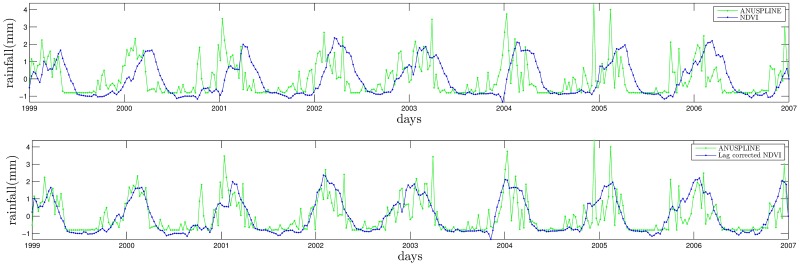
Standardized lag correction of NDVI with respect to ANUSPLINE at Chuquibambilla station.

The second step corresponds to the resizing of NDVI to a size appropriate for rainfall. As mentioned in the previous section, the ANUSPLINE outcomes are used as auxiliary data for converting NDVI into rainfall measurements. The first step in the resizing process is the standardization of both NDVI and ANUSPLINE time series at the same location. As an illustration, let us standardize the time series at Chuquibambilla station. Recall that the standardized values or *z*-score values of a time series *ρ* are obtained by the relation

$$\begin{array}{c} \rho_{standard}\left(t\right):=\frac{\rho (t)-mean}{standard\;\;deviation}. \end{array}$$

for all times. The result is presented in Fig 10 before and after correcting the time series horizontally to amount for NDVI lag (for Chuquibambilla the lag is approximately 52.5 days).

**Fig 11:**
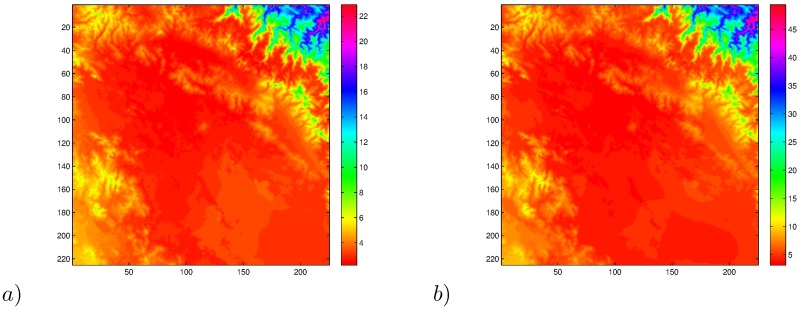
ANUSPLINE means (*a*) and standard deviation (*b*) in the area of study.

For completeness, Fig 11 show the variability of the mean and standard deviation of ANUSPLINE time series over the study area. It is worth reminding the reader that the ANUSPLINE time series only represents auxiliary information, therefore, it only provides the means and standard deviations for resizing NDVI to an appropriate size.

**Fig 12:**
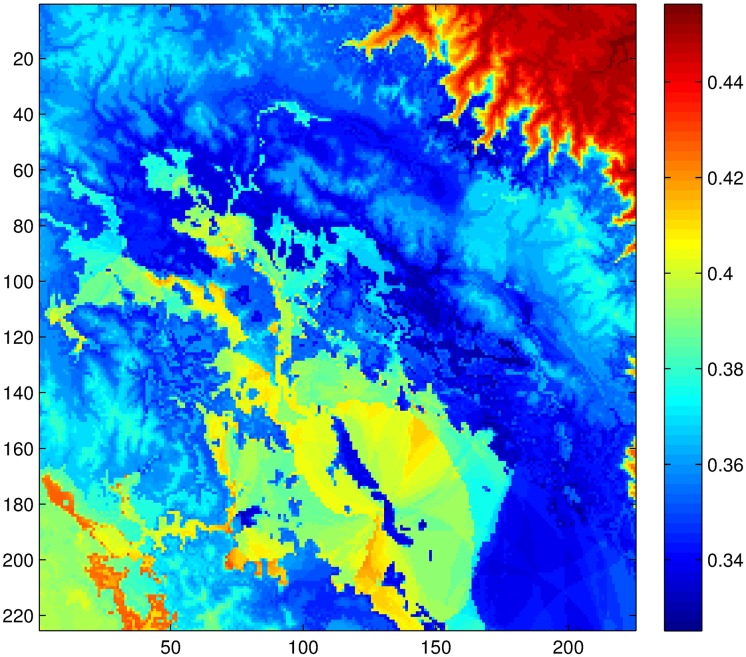
Zero probabilities of ANUSPLINE time series in the study area.

Observe that in Fig 10 the minimum value of the standardized ANUSPLINE corresponds to zero rainfall. For NDVI, it is unclear what values of NDVI correspond to zero rainfall. A pragmatic choice is to consider everything below the ANUSPLINE minimum as a value corresponding to zero rainfall. However, the cutoff value after which all values correspond to zero rain is related to the probability of zero rainfall in the NDVI time series. This zero probability can be estimated for all grid locations of the ANUSPLINE information. Therefore, the idea is to match the probability of zero rain obtained from the ANUSPLINE time series with the zero rain probability of the NDVI time series by translating the series up or down with respect to the ANUSPLINE minimum value. In this manner, one obtains the cutoff value at which NDVI corresponds to zero rainfall measurements. Fig 12 shows the zero probability on the study region. For Chuquibambilla the ANUSPLINE probability of zero is 0.1066, which is equivalent to a traslation of approximately *v* = 0.95 standardized units.

The lag corrected and vertically traslated standardized NDVI time series is transformed as

$$\begin{array}{c} NDVI_{corrected}\left(t\right):=NDVI_{standard}\left(t+lag\right)+v. \end{array}$$

**Fig 13:**
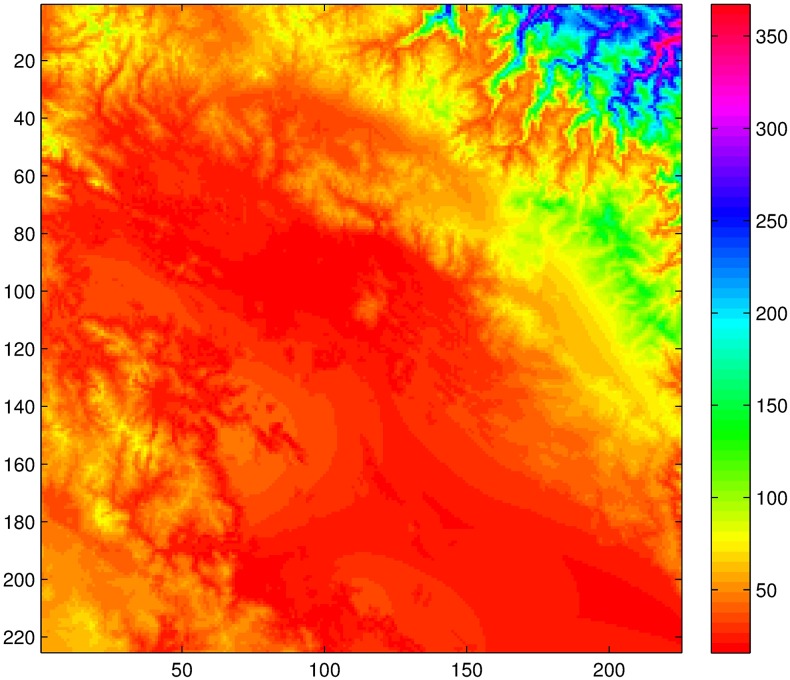
ANUSPLINE maximums in the study area.

The last step involves resizing *NDVI*_*corrected*_ using the maximum value of the corresponding ANUSPLINE time series. Fig 13, shows the maximums of ANUSPLINE time series over the study region. Since the minimum value corresponds to the value zero, the resizing procedure is equivalent to the a linear correspondence between NDVI and ANUSPLINE values.

**Fig 14:**
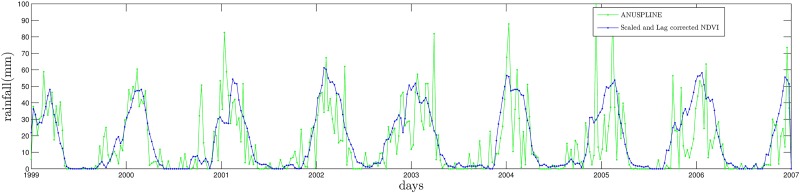
*NDVI*_*rain*_ for Chuquibambilla station.

Thus, the final equation to transform *NDVI*_*corrected*_ into rainfall measurements is

$$\begin{array}{c} NDVI_{rain}\left(t\right):=K\times NDVI_{corrected}\left(t\right), \end{array}$$

where the scale factor is defined as *K* = max(*ANUSPLINE*)/max(*NDVI*_*corrected*_). From Eq (2), one can observe that the overall NDVI transformation map is

$$\begin{array}{c} F\left(NDVI\left(t\right),lag,v\right)=K\times \left(NDVI_{standard}\left(t+lag\right)+v\right), \end{array}$$

where *lag* is an horizontal translation corresponding to the time between rainfall and the NDVI response, and *v* is the vertical translation of NDVI in order to match the zero probability of the auxiliary data. The time series *NDVI*_*rain*_ for Chuquibambilla station is given in Fig 14.

After the correction the probability of zero rainfall in *NDVI*_*corrected*_ is 0.1093, which is close to the desired 0.1066 probability of zero rainfall of the corresponding ANUSPLINE time series at the same location. It is clear from Fig 14 that NDVI is unable to detect high peaks or deep valleys of the time series corresponding to 8-day period ANUSPLINE. However, the intermittency to be introduced by the downscaling procedure will amount for sudden variations of rainfall by generating similar peaks and valleys but at the daily temporal scale as shown in the next section.

### Downscaling of *NDVI*_*rain*_ and Validation

**Fig 15:**
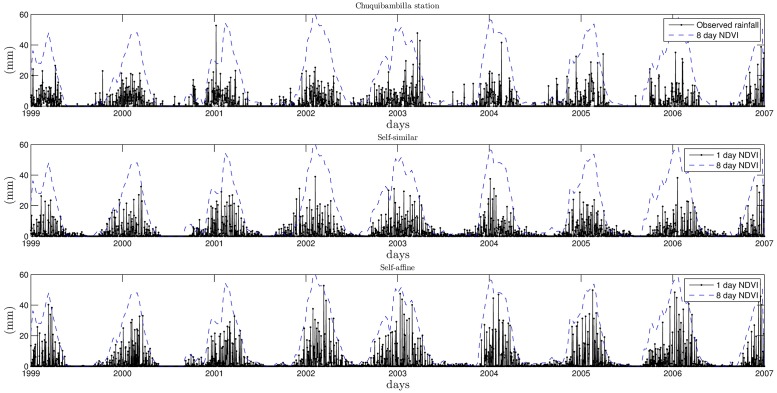
*NDVI*_*rain*_ for the self-fimilar and self-affine downscaling cases.

In this section, the downscaling procedure is applied to the *NDVI*_*rain*_ data that is at 8 day temporal resolution. The resulting 1 day resolution data is then validated against on-site measurements provided by meteorological stations in the area of study. The validation consists on a direct comparison of the statistics of the corresponding time series as well as the goodness of fit of their exceedance probabilities. In particular, the downscaling results of the *NDVI*_*rain*_ time series corresponding to the locations of 4 stations (Capachica, Chuquibambilla, Cojata and Mañazo) are presented and compared against the actual on-site rainfall values of the meteorological stations. These stations were chosen so that they are representative of the precipitation heterogeneity in the Andean plateau. Specifically, Capachica station is located in a humid area due to its influence and proximity to the Titicaca Lake. Chuquibambilla station was picked because of its surroundings semi-arid characteristics. A station in abrupt mountain terrain is Cojata station, and Mañazo station is located in a slightly mountainous arid zone. For instance, Fig 15 shows the time series for the on-site measurements, the self-similar downscaling and self-affine downscaling at Chuquibambilla station.

The statistics comparison of the on-site and generated rainfall for the self-similar and self-affine cases are given in Tables 2 and 3 for the four chosen locations, respectively.

**Table 2:** Statistics of temporal information for the self-similar case.

Station		H	Mean	Max	Skew	Q50	Q75	Var
**Capachica**	Obs	0.75	2.32	45.60	2.86	0.00	2.10	24.23
	Gen	0.77	2.15	55.73	4.19	0.00	0.73	37.38
**Chuquibambilla**	Obs	0.75	2.15	52.70	3.68	0.00	2.00	23.45
	Gen	0.68	2.07	38.97	3.61	0.12	1.75	21.33
**Cojata**	Obs	0.68	2.04	61.10	3.79	0.00	2.70	17.29
	Gen	0.72	2.22	56.89	4.26	0.00	0.79	40.61
**Mañazo**	Obs	0.77	1.93	54.00	4.05	0.00	1.50	21.64
	Gen	0.53	3.74	123.19	4.59	0.00	0.05	167.01

**Table 3:** Statistics of temporal information for the self-affine case.

Station		H	Mean	Max	Skew	Q50	Q75	Var
**Capachica**	Obs	0.75	2.32	45.60	2.86	0.00	2.10	24.23
	Gen	0.71	2.39	61.42	4.28	0.02	1.29	39.77
**Chuquibambilla**	Obs	0.75	2.15	52.70	3.68	0.00	2.00	23.45
	Gen	0.77	2.23	52.70	4.04	0.00	0.77	37.96
**Cojata**	Obs	0.68	2.04	61.10	3.79	0.00	2.70	17.29
	Gen	0.78	2.59	52.48	3.65	0.02	1.71	38.27
**Mañazo**	Obs	0.77	1.93	54.00	4.05	0.00	1.50	21.64
	Gen	0.72	2.19	62.74	4.61	0.00	0.62	46.06

Observe that the statistics of the self-affine case are closer to the statistics of the on-site measurements when compared to those of the self-similar case in the sense that they have on average an error of about 10%. For example, the Hurst exponents for the time series of on-site measurements are accurate in both self-similar and self-affine cases. However, Mañazo station improves significantly in the self-affine case with respect to the self-similar case. That is, the index moves from 0.53 to 0.72 in comparison to the 0.77 value of the on-site measurements. Similar improvements can be observed in the other statistics, where the more significant improvements are the maximums and the variances. The Hurst exponent was computed using the R/S analysis in [25–30]. Hurst exponents in the range 0.5 < *H* < 1 indicate a long-term positive autocorrelation, which implies the tendency of a high value to be followed by another high value. Also, it could indicate that the multifractal field is not conservative, which is usually handled by studying the field fluctuations [23]. Fluctuation analysis would be the concern of future research and it is outside the scope of this manuscript.

**Fig 16:**
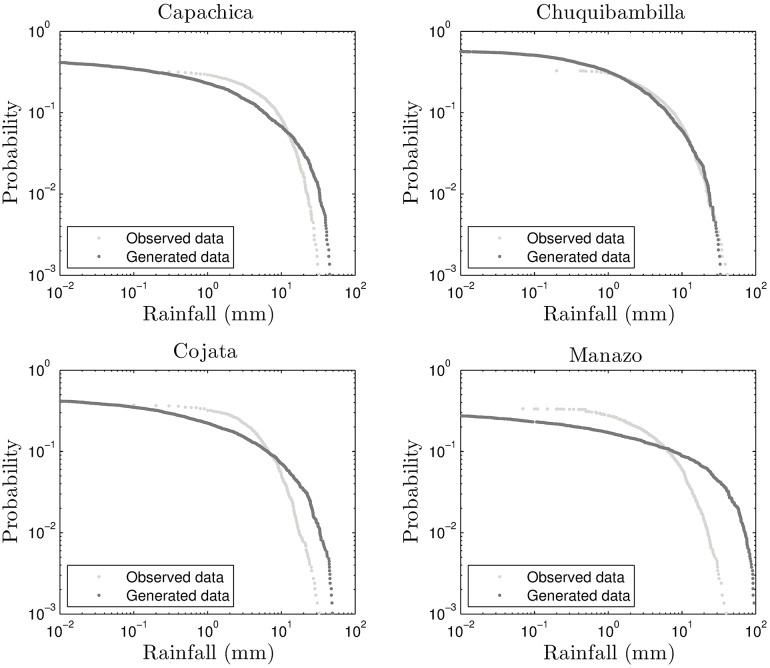
Exceedance plots for self-similar downscaling.

**Fig 17:**
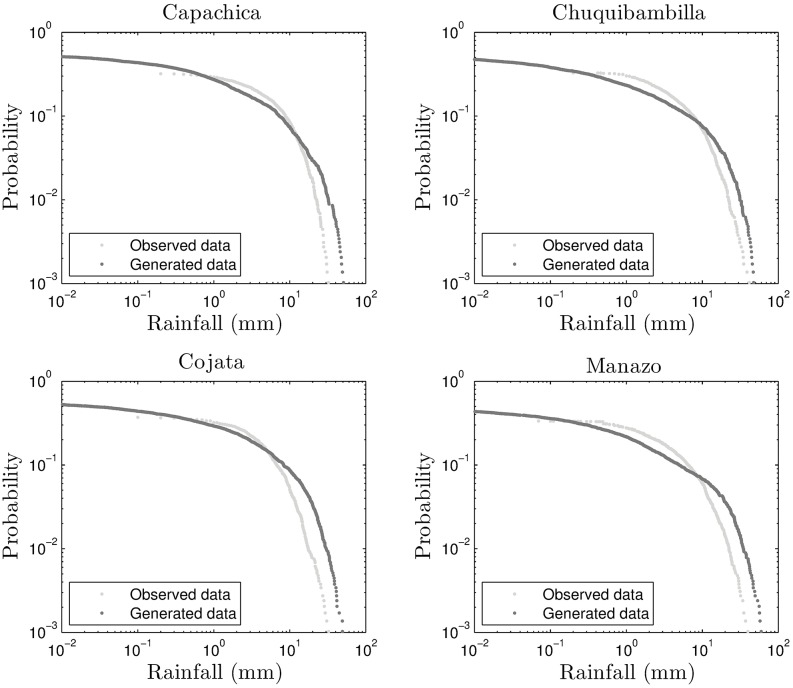
Exceedance plots for self-affine downscaling.

The exceedance probability plots of the stations under study are also a good tool for assessing the validity of the generated rainfall from NDVI information. The plots for the four stations are shown in Figs 16 and 17 for the self-similar and self-affine cases, respectively.

It is clear from these plots that the self-affine case produces a much better match with respect to the on-site measurements. This is corroborated by Tables 4 and 5. The godness of fit indicators, for the exceedance probability plots, given in the tables are: MAE (mean average error), RMSE (root mean square error), CORR (correlation coefficient), PBIAS (Percent Bias), NSE (Nash-Sutcliffe Efficiency) and RSR (ratio of RMSE to the standard deviation of the observations). These are standard indicators as described in [31]. In particular, NSE and RSR indicators are satisfactory when NSE is greater than 0.50 and the indicator RSR is around 0.80 or below, respectively.

**Table 4:** Goodness of fit for exceedance probability plots for the self-affine case.

Station	MAE	RMSE	CORR	PBIAS	NSE	RSR
**Capachica**	0.03	0.04	0.95	28.53	0.83	0.41
**Chuquibambilla**	0.01	0.03	0.97	12.74	0.92	0.28
**Cojata**	0.04	0.05	0.95	33.33	0.81	0.43
**Mañazo**	0.05	0.06	0.97	41.72	0.67	0.57

**Table 5:** Goodness of fit for exceedance probability plots for the self-affine case.

Station	MAE	RMSE	CORR	PBIAS	NSE	RSR
**Capachica**	0.02	0.03	0.95	24.32	0.87	0.36
**Chuquibambilla**	0.03	0.03	0.96	26.71	0.89	0.33
**Cojata**	0.02	0.03	0.99	26.77	0.87	0.36
**Mañazo**	0.02	0.03	0.97	15.11	0.92	0.28

**Fig 18:**
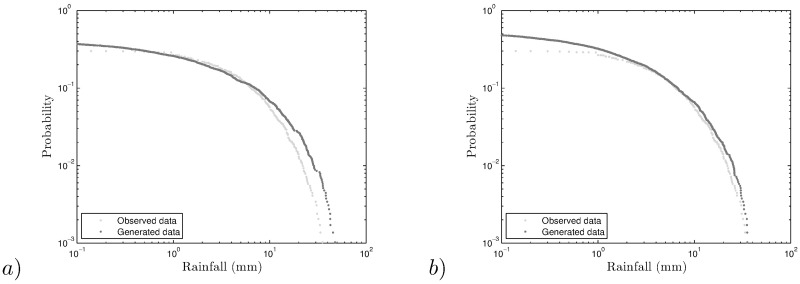
Exceedance plots for Arapa station (*a*) self-similar and (*b*) self-affine.

We want to remark that there are many locations in the area under study where the exceedance probability plots show better agreement between the observed and generated rainfall, but as mentioned before, the four chosen stations characterize the more representative regions of the Andes high plateau. As an example, the exceedance plot for Arapa station is shown in Fig 18.

**Fig 19:**
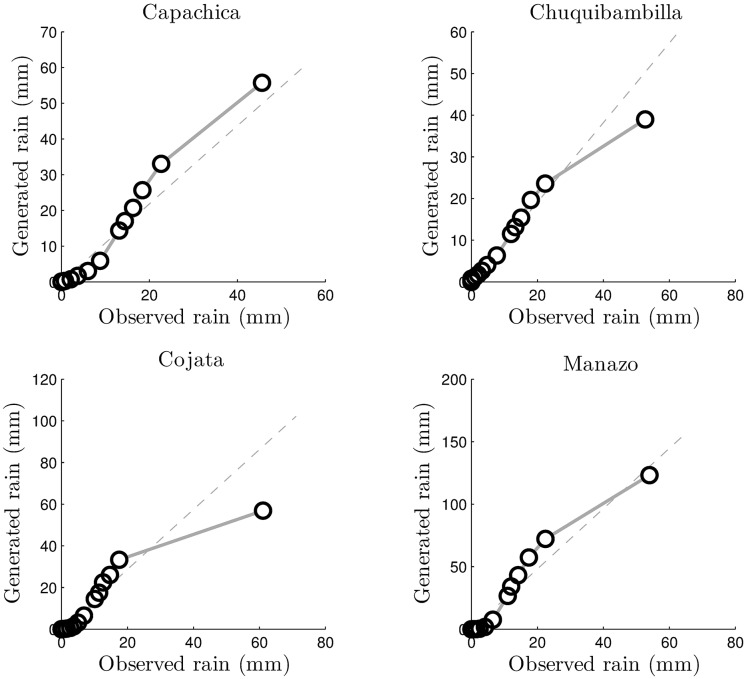
Q-Q plots for the self-similar downscaling.

**Fig 20:**
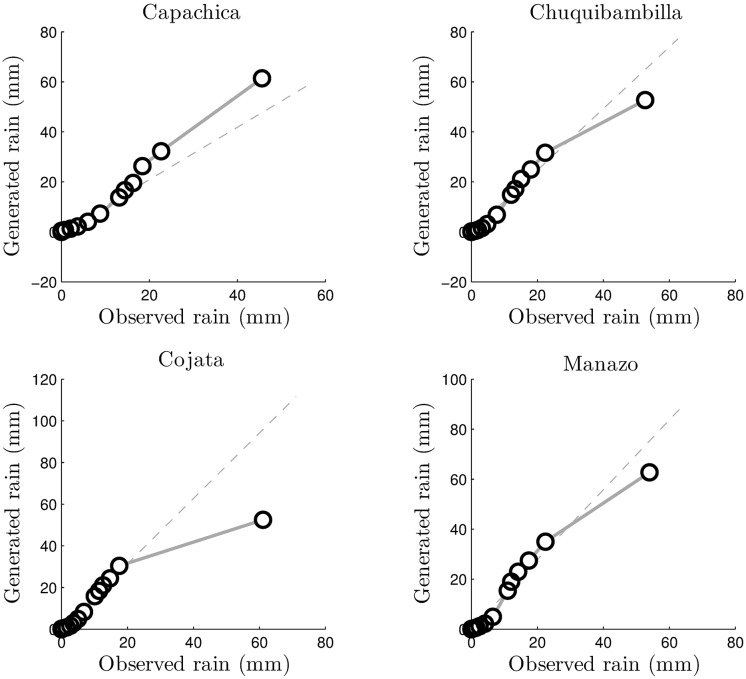
Q-Q plots for the self-affine downscaling.

The final validation tool for microcanonical downscaling in the Andes high plateau is the quantile-quantile plot (Q-Q plot). Figs 19 and 20 show such Q-Q plots for the four stations used for validation. The distributions of the observed and generated rainfall time series substantially agree in both self-similar and self-affine cases. Cojata station in particular show the largest deviation between the 99% and 100% quantile, which could be very well due to the auxiliary information provided by ANUSPLINE outcomes or the fact that Cojata is the farthest station to the east of the region of study, which amount to the rain forest influence on the area.

## Conclusion

A microcanonical downscaling technique was tested on a region of the Peruvian Andes high plateau. To amount for the lack of information in the region, NDVI informations was transformed into a 8-day period rainfall using ANUSPLINE outcomes obtained from an array of stations and an elevation map of the study area. Then, the transformed NDVI information was downscaled to a daily temporal scale. In particular, for locations representative of the heterogeneities in the area were chosen: Capachica, Chuquibambilla, Cojata and Mañazo. The generated information at these locations was then validated against on-site rainfall measurements of the corresponding meteorological stations at the same location. The generated rainfall was successfully validated even though it is known that the Andes high plateau region is challenging for such procedures due to the area’s terrain heterogeneities.

Although the procedure was successfully performed, some considerations must be taken for its application: First, the procedure is sensitive to the auxiliary information. More precisely, this auxiliary information was used for the purpose of shaping and resizing the NDVI information so that it gives an 8-day period estimation of rainfall, so wrong or altered data will affect the overall result. Second, it is clear that NDVI can only be used as a source of rainfall information in regions where the correspondence between rainfall and NDVI is valid. That is, it can only be applied in regions having precipitation ranging from 200mm to 1200mm a year. Therefore, the rainforest region (North-East corner in Fig 1) of the area under study, for example, can not be studied accurately by the procedure presented in this paper. Another limitation is that NDVI represents an aggregated measure, therefore it carries a temporal dressing of the rainfall information when downscaled to a daily scale. This of course can amount to discrepancies in the comparison of observed and generated rainfall measurements. Finally, it may be the case that a Beta distribution does not characterize the locations time series probability distribution. The case for other distributions in the *α*-stable family remains an important aspect of the downscaling procedures, however, it is outside of the scope of this manuscript.

Future work includes exploring transforming NDVI into rainfall using wavelet multi-resolution analysis, and then using temporal microcanonical downscaling to obtain daily rainfall. Also, the same methodology presented in this paper can be used for completing missing data on rainfall time series. Here the statistics and intermittency comes from the same time series (instead of auxiliary information). Therefore NDVI (through downscaling) can be employed to fill the missing data points only if the area under study is in the range allowed by the correspondence of NDVI and rainfall.

## Supporting Information

S1 Table Meteorological station data. Daily precipitation measurements for the 19 stations used in the analysis.
(XLSX)

S2 Table Meteorological station locations. Station locations within the 225 × 225 grid.
(XLSX)

S1 File NDVI data. File containing a NDVI dataset consisting of 288 (dekad) composite images (225 × 225 pixels) in Matlab format.
(MAT)
